# Polio endgame risks and the possibility of restarting the use of oral poliovirus vaccine

**DOI:** 10.1080/14760584.2018.1506333

**Published:** 2018-08-09

**Authors:** Radboud J. Duintjer Tebbens, Kimberly M. Thompson

**Affiliations:** Kid Risk, Inc., Columbus, USA

**Keywords:** Polio, eradication, dynamic modeling, disease outbreaks

## Abstract

**Introduction**: Ending all cases of poliomyelitis requires successful cessation of all oral poliovirus vaccine (OPV), but the Global Polio Eradication Initiative (GPEI) partners should consider the possibility of an OPV restart.

**Areas covered**: We review the risks of continued live poliovirus transmission after OPV cessation and characterize events that led to OPV restart in a global model that focused on identifying optimal strategies for OPV cessation and the polio endgame. Numerous different types of events that occurred since the globally coordinated cessation of serotype 2-containing OPV in 2016 highlight the possibility of continued outbreaks after homotypic OPV cessation. Modeling suggests a high risk of uncontrolled outbreaks once more than around 5,000 homotypic polio cases occur after cessation of an OPV serotype, at which point restarting OPV would become necessary to protect most populations. Current efforts to sunset the GPEI and transition its responsibilities to national governments poses risks that may limit the ability to implement management strategies needed to minimize the probability of an OPV restart.

**Expert commentary**: OPV restart remains a real possibility, but risk management choices made by the GPEI partners and national governments can reduce the risks of this low-probability but high-consequence event.

## 1. Introduction

The risks posed by different types of live poliovirus transmission change significantly over the course of the polio endgame. As long as wild polioviruses (WPVs) continue to circulate, they pose a risk of outbreaks in all areas with insufficient population immunity to transmission, particularly in the vicinity of the existing WPV reservoirs [1,2]. Oral poliovirus vaccine (OPV), the primary vaccine tool used by the Global Polio Eradication Initiative (GPEI) to stop transmission of WPVs, also comes with risks [3–7]. Specifically, OPV-using countries can expect rare cases of vaccine-associated paralytic polio (VAPP) to result from exposure of susceptible individuals (primarily children) to OPV through vaccination or close contact with OPV recipients [8]. While significantly less neurovirulent and less transmissible than homotypic WPVs, the attenuated live poliovirus strains in OPV can transmit from person to person in settings of low population immunity to transmission while evolving (i.e. losing attenuating mutations). OPV evolution and continued transmission can lead to more neurovirulent and transmissible circulating vaccine-derived polioviruses (cVDPVs) that appear to behave like homotypic WPVs [9,10]. Outbreaks of cVDPVs occur after homotypic WPV dies out and when countries fail to subsequently maintain high population immunity to transmission [11,12]. OPV offers the benefits of mucosal immunity induced by the attenuated live poliovirus infection in the intestinal tract, thereby providing not only individual immunity from paralysis but also population immunity to fecal-oral transmission. However, once WPV disappears, if OPV use continues, VAPP and cVDPVs represent the highest risks.

Globally coordinated cessation of OPV following the certification of eradication of WPV represents an essential component of the polio endgame aimed at eliminating OPV-associated risks [13]. Following the last known naturally occurring serotype 2 WPVs in 1999, in the mid-2000s, the GPEI started preferentially using serotype 1 or 3 monovalent OPV (mOPV1 or mOPV3) and bivalent OPV (bOPV, containing serotypes 1 and 3) instead of trivalent OPV (tOPV, containing all three serotypes) for supplemental immunization activities (SIAs), which led to increasing serotype 2 cVDPV (cVDPV2) outbreaks [10,14,15]. The world certified serotype 2 WPV eradication in 2015 [16], and the GPEI recognized the opportunity to globally coordinate the cessation of serotype 2-containing OPV (OPV2). OPV2 cessation occurred in late April–early May 2016 when all 155 OPV-using countries switched from tOPV to bOPV or in a few cases to only using inactivated poliovirus vaccine (IPV) [17]. Cessation of OPV use requires global coordination (i.e. synchronization) to prevent the movement of OPV-related viruses across borders from areas that continue to use OPV into those that stop [18]. A few high-income countries with limited potential for fecal-oral poliovirus transmission can maintain high enough population immunity to transmission using only IPV in their routine immunization programs [6,19–21]. Using a sequential IPV then OPV immunization schedule in countries that use OPV appears to significantly reduce VAPP [6,22]. However, IPV-induced immunity does not induce mucosal immunity in the intestinal tract or significantly reduce participation in fecal-oral transmission, and recent experience in Israel confirmed that live poliovirus transmission can occur despite very high IPV coverage in areas conducive to fecal-oral poliovirus transmission [23–25]. Following coordinated OPV cessation, complete die-out of circulating OPV-related strains can occur if population immunity to transmission is high enough everywhere that individuals infected with OPV-related viruses at the time of or shortly after coordinated cessation clear their infections without generating significant further transmission [26–28]. Surveillance suggests that OPV2-related viruses died out in almost all populations after globally coordinated OPV2 cessation. However, even if all circulating OPV-related viruses die out, in very rare instances, infected individuals with primary immunodeficiencies (PIDs) may not clear their infections (e.g. they may excrete for longer than 6 months and a very small fraction may continue to excrete for years, or even decades in one documented case[29]), and this can lead to immunodeficiency-associated vaccine-derived polioviruses (iVDPVs) [29–34]. The seeding of new iVDPVs stops at or near the time of OPV cessation, which means that the risks of reintroduction of live polioviruses into populations from iVDPV excretors should decrease with the time since OPV cessation. In the context of widespread use of OPV or populations with limited fecal-oral transmission that use IPV-only, any introduced live polioviruses, including iVDPVs, typically do not cause widespread transmission [35–37]. However, as population immunity to transmission declines following OPV cessation, any remaining iVPDVs excreted may begin to cause transmission in the surrounding communities [38]. Finally, the risk of (un)intentional releases from vaccine manufacturing sites or laboratories handling poliovirus-containing materials remains a continuing source of potential outbreaks after cessation [39,40].

We previously estimated the risks of potential reintroductions of live polioviruses over a 40-year period that began at the time of the 2013–2018 GPEI strategic plan [41]. We integrated the risk estimates with economic [42] and dynamic transmission models [43] into a global model for long-term poliovirus risk management [40]. The global model yielded estimates of aggregate health and economic outcomes of major immunization policy choices [40] assuming ideal implementation of risk management strategies, including aggressive outbreak response [44] and OPV intensification wherever needed to prevent post-cessation cVDPV outbreaks [26]. Subsequent publications using the global model considered the impact of specific risk management strategies [7,38,45] and the implications of non-synchronous OPV cessation or subsequent unauthorized OPV use [18,46,47]. However, the ideal implementation of risk management strategies prior to OPV2 cessation unfortunately did not occur universally, leading to cVDPV2 outbreaks in countries that either consciously decided not to intensify tOPV use or could not adequately intensify tOPV use due to civil unrest or poor program performance [48–50]. The quality of the outbreak response also remained sub-optimal in some cases, leading to concerns about continued transmission of the outbreak virus or the vaccine used for outbreak response seeding new cVDPV2s. Failing to rapidly control the ongoing outbreaks implies a risk of widespread serotype 2 transmission that would become increasingly difficult to control. Prior to OPV cessation, the WHO recommended introducing at least one IPV dose into routine immunization schedules in all countries [5,51], which would prevent polio cases caused by serotype 2 live polioviruses that continued to circulate after OPV2 cessation. The use of IPV does not to prevent the transmission of the OPV2-related viruses or VAPP cases caused by serotypes 1 and 3 associated with ongoing bOPV use. Due to supply constraints, countries and the GPEI did not meet the OPV2 cessation prerequisite [5,51] of introducing at least one IPV dose prior to OPV2 cessation, with some countries only beginning to receive IPV in 2018 [52]. While global population immunity to serotype 2 transmission probably remains high enough (as of early 2018) to prevent the cVDPV2 outbreaks from rapidly expanding geographically, new cohorts vaccinated against serotype 2 only with IPV can participate in transmission of OPV2-related viruses. In addition, with relatively low IPV coverage rates in many countries, some children will remain fully susceptible.

In the global model with ideal implementation of risk management strategies, post-cessation cVDPV2 outbreaks either do not occur (i.e. the risk management prevented them) or stop following an aggressive outbreak response [40,44]. However, the model suggested that if iVDPV or other live poliovirus reintroductions occurred long after OPV cessation and/or in places with conditions conducive to intense fecal-oral poliovirus transmission, then this would result in uncontrolled outbreaks and a need to restart OPV in most countries that currently use OPV [40]. Despite the ideal implementation of risk management strategies, the model suggested approximately a 5% chance of needing to restart OPV based on 1,000 stochastic iterations and a somewhat arbitrary threshold of 50,000 post-cessation polio cases used as the trigger to restart OPV use globally [38]. Recognition of the possibility of OPV restarts leads to questions about the nature of risks triggering OPV restarts, the kinetics of uncontrolled outbreaks, and strategies for risk management.

This article reviews the current epidemiological situation for serotype 2 following OPV2 cessation, provides an overview of modeling results relevant to OPV restarts to help inform risk assessments and contingency planning, and highlights risk management opportunities. To summarize the epidemiological situation following OPV2 cessation, we reviewed the experience as of April 2018, and we discuss the evidence in the context of our previously published models. To characterize the risks that may trigger OPV restarts overtime, we complement the existing GPEI post-certification strategic plan [53] with a timeline of the principal outbreak risks for each serotype based on existing global model runs [38,40]. In the context of this analysis, we highlight the limitations of the assumptions in the prior modeling with respect to the implementation of risk management strategies and the likely future polio endgame path. To examine triggers for an OPV restart, we characterize the distribution of post-cessation polio cases in the global model. Building on the previously characterized 57 stochastic iterations (out of the 1,000 total) that led to OPV restart in the global model [38], we illustrate the kinetics of outbreaks that preceded OPV restarts. We also identify priorities for future global modeling efforts that might explore the impacts of polio endgame strategies starting in 2019.

## 2. Epidemiological situation for serotype 2

Table 1 categorizes the 44 independent serotype 2 VDPV (VDPV2) events detected in the 2 years since the tOPV-bOV switch in April-May 2016, based on our subjective interpretation of limited information [48,49,54,55]. Table 1 does not include at least 2 iVDPV2s detected before the switch that still excreted after the switch (1 in Iran and 1 in the United Kingdom) [48] or a breach in containment in the Netherlands in 2017 that resulted in a WPV2-infected laboratory worker [39]. Table 1 includes one cVDPV2 event officially notified days after OPV2 cessation that identified continued transmission of a persistent cVDPV2 outbreak in a sewage sample collected in the security-compromised state of Borno, Nigeria just prior to the switch [49]. All other events represent detections of cVDPV2s (7 events), iVDPV2s (9 events), or aVDPV2s (28 events) not identified prior to the switch. Apart from the large number of events, Table 1 encompasses over 100 polio cases associated with VDPV2s and highlights the large number of possible VDPV2 events already detected in the first two years after the switch. The only possible VDPV2 events not yet detected include: (1) known iVDPV2s leading to outbreaks, (2) iVDPV2s associated with post-switch OPV2 use (either authorized mOPV2 use for outbreak response or unauthorized tOPV or mOPV2 use), (3) aVDPV2s associated with unauthorized post-switch tOPV or mOPV2 use, and (4) VDPV2s associated with a breach in containment.

**Table 1.** Categories of serotype 2 vaccine-derived poliovirus (VDPV2) events detected since the tOPV-bOPV switch and as of April, 2018, based on our subjective interpretation from limited information [48,49,54,55].

Category	Most likely source	Number of events detected in			Countries that detected the virus (number of independent events in country if not 1)	Number of AFP cases^b^
		2016^a^	2017	2018^b^		
cVDPV2	Pre-switch tOPV use	3^c^	3	0	Nigeria (2), Pakistan, Democratic Republic of the Congo (DRC), Syria, Somalia, Kenya (0)^d^	99
	Unauthorized post-switch tOPV use	0	1	0	DRC	2
	Post-switch authorized mOPV2 use	0	0	1	Nigeria	0
iVDPV2	Pre-switch tOPV use	6	3	0	Nigeria, Argentina, Egypt (3), West Bank and Gaza, Pakistan, Iran, Israel	5
aVDPV2, tail of ‘normal’ excretion distribution from OPV recipients and close contacts^e^	Pre-switch tOPV use	8	0	0	India (2), Yemen, Pakistan (3), Afghanistan, Somalia	3
	Post-switch authorized mOPV2 use	0	13	0	Nigeria (9), Pakistan (4)	0
aVDPV2, unusually long transmission chain^e^	Pre-switch tOPV use	2	1	0	Mozambique, Pakistan, India	1
	Post-switch authorized mOPV2 use	0	0	1^f^	Nigeria	0
aVDPV2 in environment, likely from immunodeficient excretor	Pre-switch tOPV use	0	1	0	Australia	0
Other aVDPV2^g^	Pre-switch tOPV use	1	0	0	Russia	0

**Abbreviations**: AFP, acute flaccid paralysis, aVDPV2, serotype 2 ambiguous vaccine-derived poliovirus; cVDPV2, serotype 2 circulating vaccine-derived poliovirus; DRC, Democratic Republic of the Congo; iVDPV2, serotype 2 immunodeficiency-associated vaccine-derived poliovirus; mOPV2, serotype 2 monovalent OPV; OPV, oral poliovirus vaccine, tOPV, trivalent OPV

^a^ Post-switch only (i.e. April or May 2016, depending on country)

^b^ Through April, 2018 (note that Somalia,, Nigeria, and DRC reported cVDPV2 cases during mid-2018)

^c^ Includes one event representing a renewed detection (environmental sample collected in March, 2016, but cVDPV2 notified days after the switch) of a persistent transmission of a cVDPV2 last detected in 2014 [49]

^d^ Kenya detected the cVDPV2 from Somalia in an environmental sample collected in March, 2018 [54]

^e^ Unusually long chain for transmission differentiated from ‘normal’ excretion distribution using a cut-off of six months since last known homotypic OPV use

^f^ Unauthorized post-switch mOPV2 or tOPV use also possible based on circumstantial information

^g^ Insufficient information available to establish nature of this event

The eight confirmed cVDPV2 events in Table 1 represent the most concerning events and reveal that at least five countries experienced challenges in attaining sufficiently high population immunity to serotype 2 transmission everywhere by the time of the switch (i.e. Nigeria, Pakistan, DRC, Syria, and Somalia). One event (i.e. the Pakistan cVDPV2) resulted from a decision to focus on interrupting WPV1 transmission without attempting to conduct sufficient tOPV SIAs. Three events involved continued detections into 2018 and therefore pose a risk for an OPV2 restart. These three events also carry important implications by demonstrating possible modes of cVDPV2 behavior after OPV2 cessation. The first represents a cVDPV2 likely associated with pre-switch tOPV use in DRC. This virus on two occasions already resulted in new cases in provinces not targeted by the outbreak response SIAs (oSIAs) up until their detection (i.e. Tanganika, Haut Katanga). This event, unlike prior post-switch cVDPV2 outbreaks in Pakistan and Nigeria, demonstrated significant geographical spread of an outbreak within a large country. The second represents the cVDPV2 that emerged in Somalia in late 2017. Although only environmental detections occurred in the first two years after the switch, Somalia reported paralytic cases of both cVDPV2 and serotype 3 cVDPV (cVDPV3) in mid-2018, and this outbreak represents the first post-switch example of documented international spread following the detection of related virus in an environmental sample in Kenya. Future observations should help determine whether the virus established transmission in Kenya. The third event is the Nigeria (state of Jigawa) cVDPV2 likely associated with post-switch mOPV2 use, which raises questions about the safety of use of the mOPV2 (i.e. the only tool currently available to stop serotype 2 outbreaks) after the switch. Prior modeling suggested that a mOPV2 outbreak response aggressive enough to stop a cVDPV2 outbreak will prevent the emergence of a new cVDPV2 from the mOPV2 used in the same population, but also suggested that much later use with low quality could lead to seeding of a new cVDPV2 (described here briefly as ‘arson’) [26]. This modeling did not consider mixing of the mOPV2 target population with neighboring areas that do not receive mOPV2. Subsequent global modeling that included the possibility of long-range exportation found a small but non-zero risk of new cVDPV2 outbreaks following mOPV2 use, but emphasized limitations in how the model accounted for mixing at the borders of the outbreak response region and point introductions of OPV-related viruses as they evolve [38,40,44]. The cVDPV2 detected in Jigawa in 2018 emerged after mOPV2 use in Jigawa and other northern Nigerian states apparently stopped a cVDPV2 in another state (i.e. Sokoto). The precise source of this new emergence remains unclear, with multiple possible causes: (1) poor coverage for the 5 mOPV2 oSIAs conducted in Jigawa since the switch, allowing spread to continue in the state, (2) poor coverage for mOPV2 oSIAs in other areas, some of which conducted only 1 or 2 mOPV2 oSIAs, allowing the mOPV2-related virus to continue to spread and evolve before returning to Jigawa, (3) exportation of mOPV2-related virus to areas that did not use mOPV2, allowing the virus to spread and evolve before returning to Jigawa, or (4) infection of a PID patient with mOPV2, who reintroduced an iVDPV2 back into the community at a time long enough after the last mOPV2 use to establish transmission. The first two explanations would represent examples of ‘arson’ associated with poor quality mOPV2 oSIAs, the third would represent an example of a new outbreak due to exported mOPV2 beyond the outbreak response areas, and the fourth would represent the first example of an iVDPV2 causing a cVDV2 outbreak. All explanations imply major challenges for the control of all future VDPV2 outbreaks. In addition, it remains possible that further detections may occur even for the outbreaks with a completed series of oSIAs and no detections in 2018, particularly in areas not reached by surveillance (e.g. Borno).

All eight cVDPV2 events in Table 1 triggered oSIAs with mOPV2 and in some cases IPV, with one very large oSIA covering most of northern Nigeria that addressed multiple independent events, but otherwise generally smaller oSIAs occurred than initially recommended [56,57]. While local conditions and risk assessments appropriately guide oSIA decisions, we caution against the risk of conducting oSIAs only in areas with known transmission because: (1) gaps and delays in information imply a possibility of unrecognized transmissions in other areas, (2) stopping transmission and preventing polio cases requires vaccinating children before they become infected [58], and (3) a reactive strategy may lead to low numbers of oSIAs conducted in different areas, which could increase the risk of seeding new cVDPV2s. Six of the eight cVDPV2 events involved use of IPV in an oSIA despite limited benefit of IPV when used in addition to mOPV2 [45,50], low cost-effectiveness [45], and insufficient IPV available for routine immunization in other countries. We recommend no further use of IPV to respond to serotype 2 events so long as mOPV2 use remains an option, unless the specifics of the event suggest that IPV may stop the outbreak without any mOPV2 use (e.g. limited iVDPV2 transmission in an under-vaccinated subpopulation with very high hygiene and sanitation).

Looking at the 27 aVDPV2 events in Table 1, we note several different types of aVDPV2 events already occurred since the tOPV-bOPV switch. The level of concern from these events varies from low (i.e. for the 21 aVDPV2 events that merely represent the tail-end of the normal pattern of excretion from tOPV or mOPV2 recipients and their close contacts) to high (i.e. viruses whose genetic age suggests unusually long transmission after a mOPV2 response). Modeling of idealized populations suggests that OPV2-related viruses eventually die out despite persisting for up to almost a year after OPV2 cessation [59], suggesting the possibility of detection of harmless aVDPVs in real populations for as long as 12 months or longer. However, an almost identical trajectory (i.e. of prevalence of OPV2-related viruses and aVDPV2s) could also represent a prelude to a cVDPV2 event. This occurred in Pakistan, where environmental surveillance detected multiple independent aVDPV2s before triggering declaration of an official cVDPV2 (i.e. after detection of a linked isolate and polio case), and also occurred before a large pre-switch cVDPV2 outbreak in Nigeria [12]. Thus, the detection of multiple independent aVDPV2s in an area beyond the typical tail of transmission following OPV2 use (e.g. 6 months) may indicate a gap in population immunity to serotype 2 transmission and a high risk of a new cVDPV2. To date, of three aVDPV2s detected more than 6 months after the switch likely tied to pre-switch tOPV use, two did not lead to further detections (i.e. one in Mozambique after two mOPV2 oSIAs, one in India without any oSIAs), while the third reflected a gap in population immunity that manifested in a contemporaneous but independent cVDPV2 event (i.e. Pakistan). In 2018, environmental surveillance in Nigeria detected another aVDPV2 (separate from the Jigawa cVDPV2) with virological age consistent with the last mOPV2 use approximately seven months earlier. Subsequent to the two-year window after OPV2 cessation represented in Table 1, this aVDPV2 revealed itself as a cVDPV2 associated with a mOPV2 oSIA following additional detections of the virus.

With the exception of the iVDPV2 detection soon after the switch in Jigawa, which occurred in the context of other cVDPV2s in Nigeria, none of the remaining eight iVDPV2s in Table 1 elicited oSIAs to date. At this point, not responding to an iVDPV2 with mOPV2 appears appropriate given the absence of any observed widespread transmission of iVDPVs in communities. The current outbreak response guidelines for 12–18 months after the switch recommend IPV vaccination of close contacts of newly detected iVDPV2s and close follow-up of the immunodeficient excretor [56]. However, it remains somewhat unclear when an iVDPV2 becomes an outbreak that warrants the same oSIA response as a cVDPV2. In the global model, we used the occurrence of enough population-wide transmission to generate the detection of a paralytic case as the trigger for oSIAs [40], but in the real world such a clear-cut distinction may not exist. For example, if a close contact of an iVDPV2 excretor with no known immunodeficiency contracts polio, then this may not yet present evidence of community transmission and may not warrant mOPV2 oSIAs. However, detection of two or more genetically linked isolates with no known direct link to the iVDPV2 excretor would constitute strong evidence of community transmission, with the vaccine and scope of the response depending on local risks factors, as with cVDPV2 outbreaks. The nine iVDPV2 excretors (i.e. five AFP cases and four non-paralytic iVDPV2 excretors) detected since the switch reflect a greater than expected proportion of the projected average prevalence of about 20 iVDPV2 excretors based on modeling [31], particularly because no system exists to detect a high fraction of non-paralytic iVDPV excretors globally. This may indicate that the fraction of OPV2-infected PID patients that develop an iVDPV2 exceeds the highly uncertain estimate of 1% used in the model [31], which we identified as a major driver of the prevalence in subsequent work [38]. Alternatively, PID survival in low- and lower middle-income countries may extend longer than estimated. Nevertheless, the absence of any newly detected iVDPV2 and reported excretors since early 2017 or iVDPV2 outbreaks provides some encouragement that iVDPV2 excretion in these countries does not last as long as expected and/or does not lead to outbreaks yet due a slower than expected decrease in population immunity to serotype 2 transmission after the switch, or a relatively low ability of iVDPV2 viruses to transmit [38].

## 3. Risks overtime by serotype

**Figure 1.**
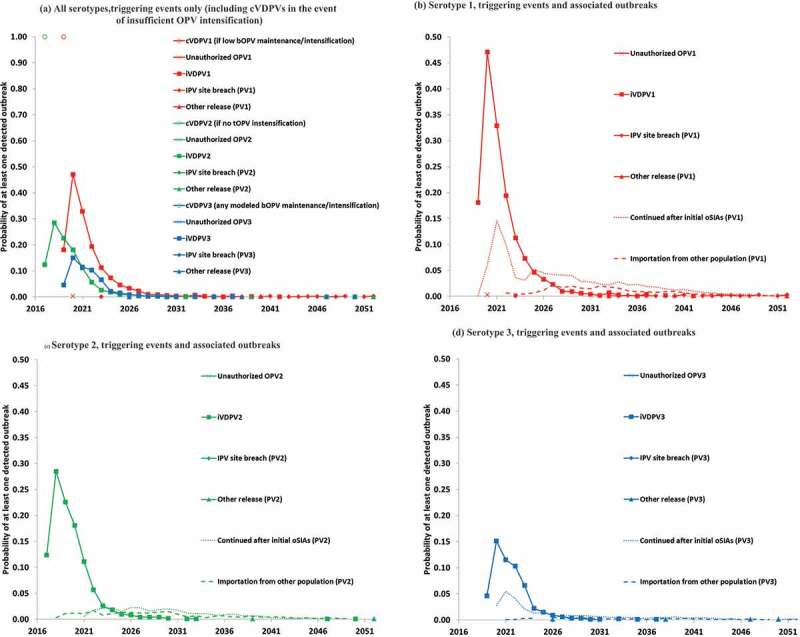
Risks overtime, by serotype and source and defined as the probability of a detected outbreak that triggers an outbreak response (i.e. oSIAs) and based on runs that assumed OPV13 cessation in 2019 [38,40]. (a) All serotypes, triggering events only (including cVDPVs in the event of insufficient OPV intensification). (b) Serotype 1, triggering events and associated outbreaks. (c) Serotype 2, triggering events and associated outbreaks. (d) Serotype 3, triggering events and associated outbreaks.

The decision to phase OPV cessation by serotype and its implementation for serotype 2 changed the relative importance of the various polio endgame risks by serotype. Complementing work by the GPEI to qualitatively map the risk overtime [53], Figure 1 summarizes the various outbreak risks overtime based on 1,000 existing stochastic iterations of the global model [38,40]. Figure 1 focuses on the probability of at least one detected outbreak, which does not reflect a measure of the generally increasing consequences of any outbreaks. We previously reported the compound risks overtime resulting from outbreak probabilities and consequences [40]. The global model assumed OPV13 cessation in 2019 followed by 5 years of continued use of one IPV dose in routine immunization in low- and lower middle-income countries and 2 or 3 IPV doses sustained in routine immunization throughout the model time horizon in upper middle- and high-income countries. With idealized implementation of OPV cessation risk management strategies and sufficient OPV intensification prior to cessation in the global model base case, no post-cessation cVDPV outbreaks emerged (i.e. deterministically a 0% chance of cVDPV outbreaks with sufficient OPV intensification prior to cessation). Based on model runs without tOPV intensification before the switch or with low bOPV SIA maintenance until OPV13 cessation, Figure 1 shows the resulting probability of 1 (i.e. 100% chance) of outbreaks in the first and second years after homotypic OPV cessation for cVDPV2 and cVDPV1, respectively [60]. No cVDPV3 outbreaks occurred for these scenarios. All other risks emerge stochastically in the global model and represent the global model base case.

Figure 1 suggests that iVDPVs dominate the non-cVDPV risks and follow different trajectories for each serotype. Specifically, with the phased OPV cessation by serotype, the probability of a detected outbreak due to iVDPVs first becomes non-zero for serotype 2 in 2017 and peaks in 2018 at approximately 0.3, followed by a decline because few iVDPV2 excretors survive in populations that cannot prevent iVDPV2 outbreaks with IPV-alone. For serotypes 1 and 3, the probability peaks in the second year following cessation of serotype 1 and 3 OPV. The delays in WPV1 eradication and serotype 1 and 3 OPV cessation will lead to larger time differences between serotypes than depicted in Figure 1. With WPV1 still circulating but no WPV3 detected for over 5 years, the rationale for switching from bOPV to mOPV1 becomes stronger. If this occurs, then it would imply even more changes in the timing of risks between serotypes. Figure 1 includes the probability of outbreaks that continue in a population despite an initial series of oSIAs (including any outbreaks seeded by the mOPV2 use) and outbreaks imported into other populations that did not yet conduct a response, both of which do not represent de novo outbreaks. These primarily reflect outbreaks initially started by iVDPVs. They reveal complex dynamics overtime as a result of temporal and geographical variability in (1) initiating outbreak probabilities, (2) population immunity to transmission, and (3) outbreak response strategies.

The probability of outbreaks from any other source (i.e. unauthorized OPV use, containment breach, other (un)intentional release from a laboratory) remain substantially smaller than the cVDPV and iVDPV outbreak probabilities (i.e. well below 1% for any given year, serotype, and source of risk) based on the assumptions of the model. However, the consequences of any of these rare events occurring long after OPV cessation in populations that can support intense fecal-oral transmission become potentially devastating, with outbreaks more difficult to control, and more likely to lead to a need to restart OPV. Thus, the ability to prevent, detect, and respond to these events even after the GPEI ceases to exist as an entity remains critical.

## 4. Post-cessation cases needed to trigger an OPV restart

**Figure 2.**
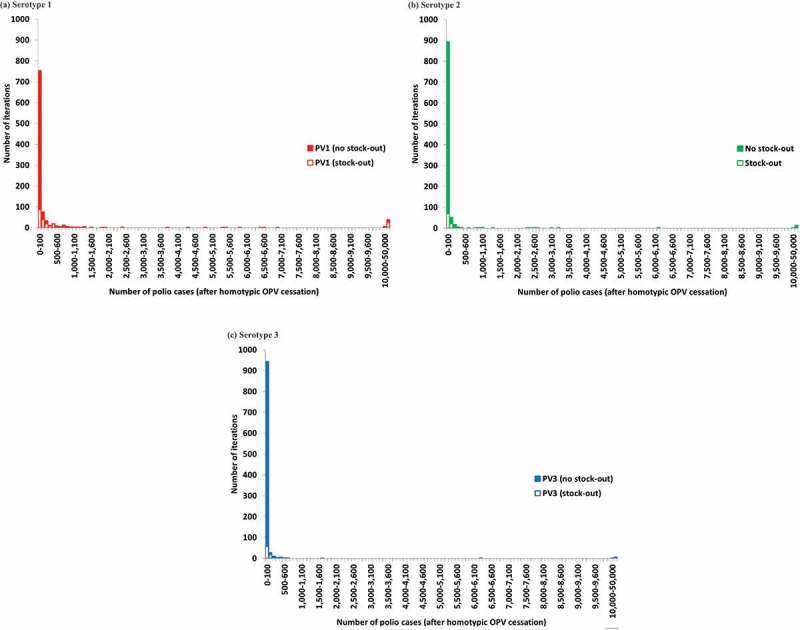
Distribution of the number of polio cases after homotypic OPV cessation in the global model base case and analysis of stock-outs (to determine whether a stock-out would occur for a given iteration, we assumed an initial and desired filled stockpile level of 100 million doses with 400 million bulk doses and a one-year filling time.). (a) Serotype 1. (b) Serotype 2. (c) Serotype 3.

To inform the possible outcomes of the risks presented in Figure 1, Figure 2 provides the distribution of the number of polio cases resulting from all of the outbreaks for each serotype, which takes into account population immunity to transmission after OPV cessation, aggressive outbreak response, and potential transmission between populations [40]. Figure 2 illustrates the extremely long tail of possible numbers of polio cases. For each serotype, the vast majority of iterations resulted in less than 100 post-cessation polio cases (i.e. 75%, 89%, and 94% for serotypes 1, 2, and 3, respectively), while very few led to uncontrolled outbreaks with more than 50,000 polio cases for which the model assumed an OPV restart would occur (i.e. 3.9%, 1.4%, and 0.6% for serotypes 1, 2, and 3, respectively). We emphasize that the implementation of many risk management policies did not occur as optimally as we modeled them in 2015 and that the distribution would change if we included more recent information. For example, for serotype 2, already over 100 cVDPV2 cases occurred compared to none in the global model base case. Furthermore, the global model base case assumed no constraints on the amount of mOPV or IPV available for outbreak response. Figure 2 breaks down the results by iteration for which a mOPV2 stock-out would occur for an initial mOPV stockpile of 400 million bulk and 100 million finished doses with a 1-year filling time [7,44]. When we consider this constraint on the stockpile size, Figure 2 shows that even some of the iterations with relatively few cases would lead to a stock-out and therefore represent an elevated OPV restart risk.

**Figure 3.**
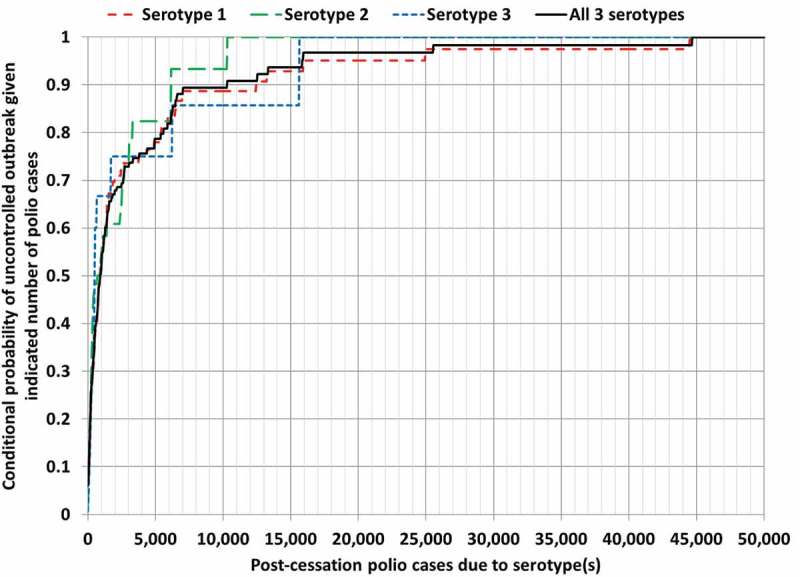
Conditional probability of uncontrolled outbreaks (i.e. more than 50,000 polio cases, which triggers an OPV restart in the global model [38]) as a function of the number of polio cases, based on the results from Figure 2.

Figure 2 illustrates the rarity of runs with many cases (e.g. more than 1,000) for which all outbreaks ultimately get controlled and avoid an OPV restart (i.e. fewer than 50,000 cases). Figure 3 shows the corresponding conditional probability of uncontrolled outbreaks (defined as iterations with 50,000 or more cases, which would lead to an OPV restart in the model) given the occurrence of the indicated number of polio cases. Figure 3 suggests that the probability of uncontrolled outbreaks remains less than 50% for each serotype as long as fewer than 1,000 polio cases occur, but that it increases steeply between 1,000 and 5,000 polio cases, with around 80% chance of an eventual OPV restart if 5,000 cases occur for any given serotype.

## 5. Kinetics of OPV restarts

**Figure 4.**
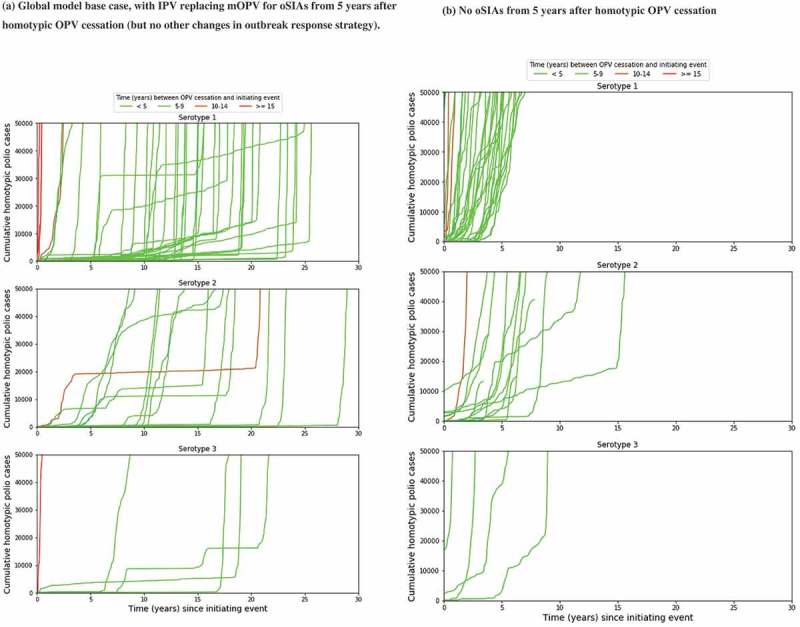
Kinetics of 57 global model iterations that resulted in an OPV restart for different categories of times between homotypic OPV cessation and the initiating event that eventually triggers the OPV restart. (a) Global model base case, with IPV replacing mOPV for oSIAs from 5 years after homotypic OPV cessation (but no other changes in outbreak response strategy). (b) No oSIAs from 5 years after homotypic OPV cessation.

Figure 4 shows the kinetics of the 57 OPV restarts out of 1,000 global model base case iterations [38,40]. The global model base case assumed that 5 years after homotypic OPV cessation, any oSIAs would switch from using mOPV to IPV, with the target age groups increasing with time since OPV cessation and no other changes [44]. While this strategy typically controlled outbreaks in settings with low fecal-oral transmission, for the 57 OPV restart iterations it merely resulted in repeated series of IPV oSIAs requiring unrealistically large numbers of IPV doses [7,44]. As shown in Figure 4(a), this often resulted in containment of the outbreak in the original outbreak population for many years until eventually a long-range exportation of the outbreak virus led to explosive propagation of the outbreak to all vulnerable populations. While the slow kinetics may suggest multiple years available to restart production and prepare for an OPV restart, Figure 4(b) more realistically shows the much faster kinetics when assuming no availability of IPV from an enormous stockpile to conduct the repeated IPV oSIAs. This suggests a much shorter time to prepare for an OPV restart, particularly for serotype 1. The shading in Figure 4(a-b) reflects increasingly long times between homotypic OPV cessation and the initiating event (i.e. the introductions of live poliovirus into the population that restarts uncontrolled transmission) for colors varying between light green and red, respectively. This illustrates that later introductions tend to result in an OPV restart much faster compared to virus introduced soon after OPV cessation.

## 6. Factors affecting current OPV restart risk

Table 2 qualitatively summarizes the impacts of numerous strategies identified in prior work as critical to minimize the risk of needing to restart OPV [7,38,40,44,46,61], and the internal (national) and external (GPEI or post-GPEI international donors) resources required for them. Table 2 aims to complement the draft post-certification strategy developed by the GPEI [53].

**Table 2.** Recommended strategies to minimize the risk of an OPV restart and qualitative impacts and resource needs.

Risk management strategy	Impacts	Level of internal (national) resources required	Level of external (GPEI or post-GPE international donors) resources required
Maintain high OPV coverage prior to OPV cessation	++++ serotype-specific population immunity to transmission prior to OPV cessation significantly impacts cVDPV risks and probability of OPV restart	+ for countries that currently conduct OPV SIAs	++ for countries that currently conduct OPV SIAs with external support
Coordinate OPV cessation globally	+++ critical to minimize post-OPV cessation cVDPV risks	+ for countries that currently use OPV	+ for coordination and monitoring
Perform aggressive outbreak response using mOPV in near-term after OPV cessation	+++ essential to stop transmission of live polioviruses post-OPV cessation to prevent OPV restart	++ for countries with post-OPV cessation outbreaks	+ for countries that currently conduct OPV SIAs with external support
Perform aggressive outbreak response using available resources in long-term after OPV cessation	+++ essential to stop transmission of live polioviruses post-OPV cessation to prevent OPV restart	++ for countries with post-OPV cessation outbreaks	+ for countries that currently conduct OPV SIAs with external support
Outbreak response poliovirus vaccine global stockpiles	+++ essential to support outbreak response activities		+ for OPV +++ for IPV long-term
Continue AFP surveillance	+++ essential through OPV-cessation	+ for countries that use AFP	++ for supporting the Global Polio Laboratory Network
Continue or add environmental surveillance (ES)	+ may help with confidence about no live poliovirus transmission, impact depends on design of the system	++ for countries that choose to include ES	++ for coordination and support of countries that require external resources
IPV use after cessation of last OPV serotype	+++ essential in countries that continue to produce poliovirus vaccines, store live polioviruses, and/or sustain potential iVDPVs + provides limited insurance that protects vaccinated individuals	++++ for 2-dose schedule	++++ for countries that require external support
Ensure containment	+++ essential to prevent the reintroduction of live polioviruses after OPV-cessation	+ for most countries +++ for countries that choose to maintain stocks of live polioviruses	++ for global coordination
Development of polio antiviral drugs	? depends on characteristics of products actually developed (e.g. efficacy, cost, ease of delivery, etc.)		++ to finish development
Prevention of iVDPV outbreaks by screening for PIDs	? depends on ability to detect iVDPVs and efficacy of polio antiviral drugs	++ for countries with potential iVDPV excretors	++ for countries that require external support
Develop new OPV and or IPV seed strains	? depends on characteristics of products actually developed		+++ for development

The GPEI did not meet several of the prerequisites for OPV2 cessation [5], and consequently we see many differences between the modeled idealized implementation of OPV2 cessation and what actually happened that impact OPV restart risks. Most importantly, the cVDPV2 outbreaks in the two years following OPV2 cessation exposed serious program performance challenges in security-compromised areas and various parts of sub-Saharan Africa. However, the absence of cVDPV2s to date anywhere else in the world represents a major, under-appreciated success. Prior to OPV2 cessation, no direct evidence existed that OPV2 cessation could succeed beyond some observations of die out in limited settings (i.e. upper middle- and high-income countries with high-quality immunization programs). Fortunately, widespread cVDPV2 outbreaks in low- and lower middle-income countries did not occur to date owing to good immunization programs and adequate tOPV intensification in most high-risk areas. Nevertheless, the ongoing cVDPV2 transmission in multiple African countries two years after the switch represents a major challenge for the GPEI, which requires aggressive control with mOPV2, despite its risks, because no other viable option exists. To prepare for the possibility that the mOPV2 used now fails to stop cVDPV2 transmission or results in further new cVDPV2s, the GPEI must develop its contingency plan now for the reintroduction of tOPV in most countries.

With respect to OPV13 cessation, we expect similar or higher OPV coverage required to prevent cVDPV1 outbreaks than needed to prevent cVDPV2 outbreaks after the switch [46]. If GPEI resources and SIAs decrease, then all countries identified as weak links following OPV2 cessation would likely also face elevated risks associated with OPV13 cessation. Thus, maintaining high-quality SIAs in these populations will remain critical until OPV13 cessation, with additional pre-cessation SIAs likely needed in countries with good programs but high poliovirus transmissibility (e.g. India) [46]. Moreover, ensuring IPV supply for routine immunization remains critical to limit iVDPV outbreak risks and protect children from post-cessation paralysis due to cVDPVs. Furthermore, the GPEI should give serious consideration to a bOPV-mOPV1 switch in light of the possibility of further delays of WPV1 eradication to stop creating OPV3-related risks (i.e. serotype 3 VAPP, iVDPV, and cVDPV). With the GPEI using mOPV1 in some SIAs in Pakistan in 2018 and continued signs of transmission of WPV1 in Pakistan and Afghanistan raising concerns about the ability to stop WPV1 transmission globally by 2019, the urgency to consider coordinated OPV3 cessation emerges, particularly as OPV producers increasingly plan to leave the market.

The post-cessation outbreak responses to date generally remained less aggressive than we assumed, particularly with respect to scope (except for one large oSIA in Nigeria). So far, it appears that this less aggressive response sufficed to stop most cVDPV2 outbreaks, although it remains too early to declare this with confidence. Moreover, the DRC outbreak appears to continue spreading geographically beyond the areas targeted by multiple outbreak response rounds. High coverage remains critical to minimize the risk, and the number of oSIAs with mOPV should increase in areas not capable of attaining high oSIA coverage.

Ideally, AFP surveillance should continue at a level that can reliably detect at least every second or third AFP case caused by live polioviruses anywhere in the world, strategically supplemented by environmental surveillance where it adds value. Although IPV use prevents AFP in successfully vaccinated children, AFP surveillance remains the only comprehensive system to detected polioviruses wherever susceptible children exist (i.e. either unvaccinated or those who fail to seroconvert to one or more IPV doses). IPV use in routine immunization remains critical to reduce the risk from iVDPVs, particularly in upper middle- and high-income settings with the highest iVDPV prevalence and the highest impact of IPV on population immunity to serotype 2 transmission. In contrast, for low and lower middle-income countries, the risk of producing IPV using transmissible seed strains (i.e. WPV or OPV) may outweigh the benefits of continued IPV use for more than 5 to 10 years after OPV2 cessation [40].

The risks of poliovirus releases or other laboratories highlight the need to focus additional efforts on containment now that the post-OPV era is well underway for serotype 2 [4,38,39,62,63]. In this respect, work to develop IPV seed strains that do not replicate or revert to a neurovirulent and transmissible form offers the potential for safer IPV production in otherwise high-risk countries. However, the cost-effectiveness and willingness to self-fund IPV use for long after OPV cessation (if no major outbreaks occur) remains questionable [40,45]. In addition, the long-term risks, costs, and benefits of IPV use remains uncertain, and future analyses may not support the current recommendation of at least 2 IPV doses for at least 10 years after cessation of the last OPV serotype [64], which the WHO recommended in the absence of any economic or risk analyses. Nevertheless, with the risks of mOPV2 use long after OPV cessation, a minimal IPV stockpile is needed in addition to mOPV stockpiles [7,44] to respond to any long-term introductions in low or moderate transmission risk settings (e.g. iVDPVs), and to potentially reduce paralytic cases following an introduction into higher transmission risk settings.

Antivirals offer an ability to significantly reduce iVDPV risks and yield up to $1.5 billion in expected net benefits, but only if PID surveillance can identify a high fraction of non-paralytic iVDPV excretors [38]. The costs and benefits of efforts to screen PIDs for live poliovirus excretion and potentially treat them to stop their excretion using polio antiviral drugs remain important areas for further research. Finally, a new OPV vaccine without the risks of the current OPV (i.e. without VAPP and/or VDPVs) could provide a solution for the current lack of an outbreak response strategy that remains both safe and effective. A new and safer OPV could make the prospect of restarting OPV dramatically less daunting, although we recognize the many challenges associated with developing, testing, licensing, producing, and reintroducing a new vaccine against a virus last reported in naturally occurring form in the previous century. Future analyses should help to further clarify the risks discussed in Table 2 and ultimately provide quantitative estimates for the different strategies. Ongoing research may also yield other risk management opportunities [65].

## 7. Expert commentary

Despite recognition of the importance of continued coordination of global risk management efforts to achieve the ultimate goals of polio eradication and a successful OPV endgame, significant risks remain. While earlier modeling assumed the existence of coordinated global risk management activities, dissolution of the GPEI appears likely prior to the completion of coordinated global cessation of serotype 1 OPV. More significantly, the GPEI and countries failed to meet the pre-requisites established for OPV2 cessation, and ongoing transmission of serotype 2 polioviruses represents a threat to the ultimate objective of the 1988 resolution of ending all poliomyelitis [66]. As part of ongoing GPEI transition activities, some countries already show diminished capacities for performing both preventive and reactive activities required to successfully navigate the polio endgame.

The nature of the risks continues to evolve, along with our understanding about them. However, the risks represent significant enough threats to polio eradication that continued investment in risk management will be required, and the GPEI needs to ensure a smooth transition to parties who will manage the critical responsibilities throughout the remainder of the polio endgame.

Even with the best risk management, OPV restart remains a non-zero risk. This review emphasizes that the probability of OPV restart depends on risk management choices made by national governments, the GPEI, and its successors. Stockpiles of OPV for outbreak response available shortly after OPV cessation would not prove sufficient to supply OPV for the populations that would need it in the event of an OPV restart. Thus, the GPEI partners should develop strategies to rapidly restart production of appropriate poliovirus vaccines in the event of uncontrolled outbreaks. In the context of current research and development, this effort could focus on a promising potential new OPV (nOPV) strain, and it could involve some use of OPV produced by IPV manufacturers using Sabin strains if those manufacturers licensed their OPV for such use.

Reflecting on the actual experience with OPV2 cessation and the lack of a strategic plan or budget for the polio eradication endgame after 2018 (or 2019), we remain concerned that financial and coordination risks may emerge as the most significant threats to successful polio eradication. Eradication represents an unforgiving permanent prevention goal, and its achievement will require successful management for the foreseeable future.

## 8. Five-year view

In 2012, we anticipated that by now, the uncertainties about potential low-cost IPV options would get resolved and make the choice to switch to IPV relatively easier [5]. Unfortunately, as of 2018, IPV costs remain relatively high, and uncertainty remains about the likelihood of significant future decreases of IPV costs. After the eradication of WPVs, we anticipated that even with a low-cost IPV option, some countries might prefer to use their scarce resources for other health interventions. However, the current WHO SAGE recommendation suggests that after global OPV withdrawal, all countries should include ‘at least 2 doses of IPV in their routine immunization schedule’ for at least 10 years after OPV withdrawal [64]. This recommendation implies significant costs for the polio endgame, and it will make the overall cost of polio eradication significantly higher than assumed by prior analyses [40,67]. Given that SAGE made its recommendation without any consideration of cost or cost-effectiveness, we anticipate that in the next 5 years, national governments will evaluate their commitment to continued IPV vaccination, and this may lead to reconsideration of the SAGE IPV recommendation.

As the GPEI partners put forward the Post-Certification Strategy [53], this work should support ongoing discussions about post-certification risks, and the costs and benefits of the different options for managing post-certification risks. Over the next five years, we anticipate that the GPEI may dissolve and the GPEI partners will establish whatever structure and entities will exist to manage polio endgame risks. We expect that during this time, the GPEI partners will also consider the planning for OPV restart. We hope that the discussions will better consider the impacts of choices made with respect to the economic impacts.

In 2014, we anticipated that by 2018, global health leaders would certify the world as free of wild poliovirus type 3, and we hoped for the successful elimination of all WPVs and cVPDV2s [6]. As discussed in this review, certification of WPV2 eradication and coordinated OPV2 cessation occurred, but cVDPV2s continue to circulate, and the certification of WPV3 eradication has not occurred. The delay of disruption of transmission of WPV1 eradication continues to delay the entire polio endgame, and the costs of polio eradication continue to increase.

In 2017, we expected that within the next few years, global population immunity to serotype 2 transmission would continue to decrease and the size of cohorts with no serotype 2 vaccine protection would accumulate [7]. Serotype 2 population immunity to transmission is declining, and although the use of IPV may protect cohorts born since OPV2 cessation from paralysis upon infection with a serotype 2 live poliovirus, IPV will not prevent participation in transmission. We expect that it will continue to decline, and during the next 5 years, countries will either stop all remaining chains of transmission of serotype 2 live polioviruses or begin plans to restart serotype 2-containing OPV use. We remain concerned that outbreak response efforts following OPV cessation may not stop serotype 2 live poliovirus transmission.

We continue to expect significant changes in the poliovirus vaccine market over the next five years and to hope that efforts to develop less expensive and better poliovirus vaccines will lead to better options. We expect that the GPEI partners will develop and maintain appropriate poliovirus vaccine stockpiles (i.e. OPV, IPV). However, as the GPEI dissolves, the institutional support for these efforts remains uncertain.

## Key issues

- The polio endgame will continue to require active risk management and use of resources
- Efforts to transition the GPEI to a post-certification strategy pose risks associated with the potential for insufficient resources and coordination
- The need to restart OPV remains a real possibility, although the probability remains low so long as countries and the GPEI partners manage risks
- Failing to manage polio endgame risks threatens the success of polio eradication
- The GPEI partners should prepare and plan for the possible need to restart OPV
